# Engineering Stem Cell Factor Ligands with Different c-Kit Agonistic Potencies

**DOI:** 10.3390/molecules25204850

**Published:** 2020-10-21

**Authors:** Tal Tilayov, Tal Hingaly, Yariv Greenshpan, Shira Cohen, Barak Akabayov, Roi Gazit, Niv Papo

**Affiliations:** 1 Avram and Stella Goldstein-Goren Department of Biotechnology Engineering and the National Institute of Biotechnology in the Negev, Ben-Gurion University of the Negev, Beer-Sheva 8410501, Israel; taltil@gmail.com (T.T.); talhingaly@gmail.com (T.H.); 2 Shraga Segal Department of Microbiology, Immunology and Genetics and the National Institute of Biotechnology in the Negev, Ben-Gurion University of the Negev, Beer-Sheva 8410501, Israel; yarivg@post.bgu.ac.il (Y.G.); gazitroi@bgu.ac.il (R.G.); 3 Department of Chemistry and the National Institute for Biotechnology in the Negev (NIBN), Ben-Gurion University of the Negev, Beer-Sheva 8410501, Israel; shiracoh@post.bgu.ac.il (S.C.); akabayov@bgu.ac.il (B.A.)

**Keywords:** receptor tyrosine kinases, protein-protein interactions, protein engineering, directed evolution, angiogenesis, binding affinity, agonistic activity

## Abstract

Receptor tyrosine kinases (RTKs) are major players in signal transduction, regulating cellular activities in both normal regeneration and malignancy. Thus, many RTKs, c-Kit among them, play key roles in the function of both normal and neoplastic cells, and as such constitute attractive targets for therapeutic intervention. We thus sought to manipulate the self-association of stem cell factor (SCF), the cognate ligand of c-Kit, and hence its suboptimal affinity and activation potency for c-Kit. To this end, we used directed evolution to engineer SCF variants having different c-Kit activation potencies. Our yeast-displayed SCF mutant (SCF_M_) library screens identified altered dimerization potential and increased affinity for c-Kit by specific SCF-variants. We demonstrated the delicate balance between SCF homo-dimerization, c-Kit binding, and agonistic potencies by structural studies, in vitro binding assays and a functional angiogenesis assay. Importantly, our findings showed that a monomeric SCF variant exhibited superior agonistic potency vs. the wild-type SCF protein and vs. other high-affinity dimeric SCF variants. Our data showed that action of the monomeric ligands in binding to the RTK monomers and inducing receptor dimerization and hence activation was superior to that of the wild-type dimeric ligand, which has a higher affinity to RTK dimers but a lower activation potential. The findings of this study on the binding and c-Kit activation of engineered SCF variants thus provides insights into the structure–function dynamics of ligands and RTKs.

## 1. Introduction

Receptor tyrosine kinases (RTKs) are major players in signal transduction, regulating cellular activities according to the availability and potency of their cognate ligands. RTKs and their ligands have thus been extensively studied with the dual aims of elucidating their biochemical properties and finding the means to manipulate them for clinical purposes [1,2,3,4]. However, despite the work that has been done to date, the delicate balance of RTK-ligand interactions and functional activation is not yet fully understood. It remains difficult to predict the relationship between ligand dimerization, specific RTK-ligand affinity, and functional activation potency. It has, for example, been shown for vascular endothelial growth factor (VEGF), human growth hormone (hGH), and macrophage colony stimulating factor (M-CSF) that activation of their cognate RTKs is balanced between self-dimerization and RTK affinity [5,6,7,8,9,10]. Thus, ligand ‘fitness’ (optimal dimerization and binding affinity) is usually not optimal, most probably due to inherent regulatory requirements for dynamic and evolutionary tuning [11,12,13,14].

Among the early attempts to engineer the conversion of RTK agonistic ligands into antagonists were those that focused on VEGF, hGH, M-CSF and stem cell factor (SCF) [11,12,13]. For example, abolishing ligand dimerization aimed to generate monomers that would act as receptor antagonists [15]. However, this approach was impeded by the diminished affinity for the RTK of the monomeric ligand relative to the dimeric ligand, which limited the therapeutic usage of such monomers [11,15,16]. Current methodologies aimed at generating potent RTK antagonists and agonists are thus based on protein engineering designed to modify ligands by changing both their affinities [17] and their self-dimerization status [14]. However, since affinity and self-dimerization are not mutually exclusive, the engineering of optimized protein-based antagonists or agonists must find the delicate balance between improved binding affinity and impaired self-dimerization [18]. In practical terms, combinatorial site-directed engineering of both ligand-ligand and ligand-receptor interactions provides the means to generate improved therapeutic mediators and to gain insights into the dynamics of RTK-ligand interactions.

A prototypic RTK-ligand interaction that has been extensively studied and that has generated important milestones in the field is the c-Kit/SCF interaction [19], which plays a key role in the regulation of epithelial, endothelial, neuronal, and hematopoietic stem-progenitor cells (HSPCs) [20,21,22]. For example, dysregulation of c-Kit/SCF signaling and gain-of-function c-Kit mutations have been implicated in different cancers [23,24,25,26,27,28], including thyroid carcinoma, oncocytic intraductal papillary mucinous neoplasms (IPMNs) of the pancreas, and lung cancer [29,30,31]. To understand c-Kit/SCF signaling, it is necessary to take a brief look at the two partners and at what is currently known about them. SCF is a four-helix bundle-type small protein that forms non-covalent homo-dimers, which may be membrane-anchored or soluble, depending on alternative RNA splicing and proteolytic processing [13,32]. The functional core of SCF is the N-terminal domain, which includes the dimerization interface and the portions of the molecule that bind to c-Kit [33]. Following bivalent binding of an SCF-dimer to a c-Kit monomer, the receptor dimerizes with another c-Kit protein, thereby bringing the two intracellular kinase domains into proximity and allowing them to phosphorylate one another to promote the signal transduction cascade [34,35,36]. As is the case for other growth factors, such as platelet-derived growth factor (PDGF) and VEGF [37], SCF dimerization is a prerequisite for the proper activation of c-Kit [35]. Indeed, mutated SCF showed reduced c-Kit activation and reduced mitogenic activity in cells, due to impaired SCF self-dimerization, reduced c-Kit affinity, or both [13]. Although the c-Kit receptor activation process has been studied extensively using monoclonal antibodies [38], recombinant protein receptors [35,39], and small molecule kinase inhibitors [36,40,41,42,43,44], the correlations between SCF concentration, SCF dimerization, SCF-c-Kit binding affinity and c-Kit receptor activation are not always fully understood. A particular problem is that most studies showing that wild-type SCF (SCF_WT_) is highly potent in activating c-Kit have been conducted with very low concentrations (low nanomolar range) of SCF_WT_, whereas effective SCF tissue concentrations are much higher (in the micromolar range), especially when SCF is intended for use as a therapeutic. To address this problem, it is necessary to generate and study SCF-derived proteins with altered self-dimerization properties and altered affinity for c-Kit that confer, at high concentrations, a superior agonistic function in comparison to SCF_WT_. Such engineered SCF-derived proteins will not only provide insights into the molecular mechanisms that mediate receptor activation but will also serve as a basis for further manipulations of therapeutic value.

In this study, we combined rational and combinatorial engineering approaches for transforming dimeric SCF into ligands with different agonistic potencies at high SCF concentrations. Specifically, we engineered variants with a reduced dimerization potential and an increased affinity for c-Kit. To this end, we screened random mutagenesis SCF monomer (SCF_M_) libraries to identify SCF_M_ variants with different self-association properties and different affinities for c-Kit and hence with different degrees of activation of c-Kit. Importantly, despite the parental SCF_M_ being the only monomer variant that binds c-Kit and despite its lower affinity for c-Kit vs. the wild-type dimeric SCF (SCF_WT_) and vs. the other SCF_M_ variants, SCF_M_ was superior to SCF_WT_ and to the other SCF_M_ variants in activating c-Kit. Our data shows that the impairment of SCF dimerization may increase the local SCF concentration near c-Kit and thereby induce enhanced dimerization and activation of the receptor. Studying a collection of SCF_M_ variants with optimized functions enabled us both to identify preferential agonists and also to study the relationship between ligand dimerization and c-Kit receptor activation. We suggest that potent activators for other RTKs could be generated by applying a similar structure-based library screening approach, which would, in turn, facilitate further investigation of the mechanisms underlying the activation of RTKs.

## 2. Results

### 2.1. Sorting of SCF_M_ Libraries for Variants with a High Affinity for c-Kit

**Figure 1:**
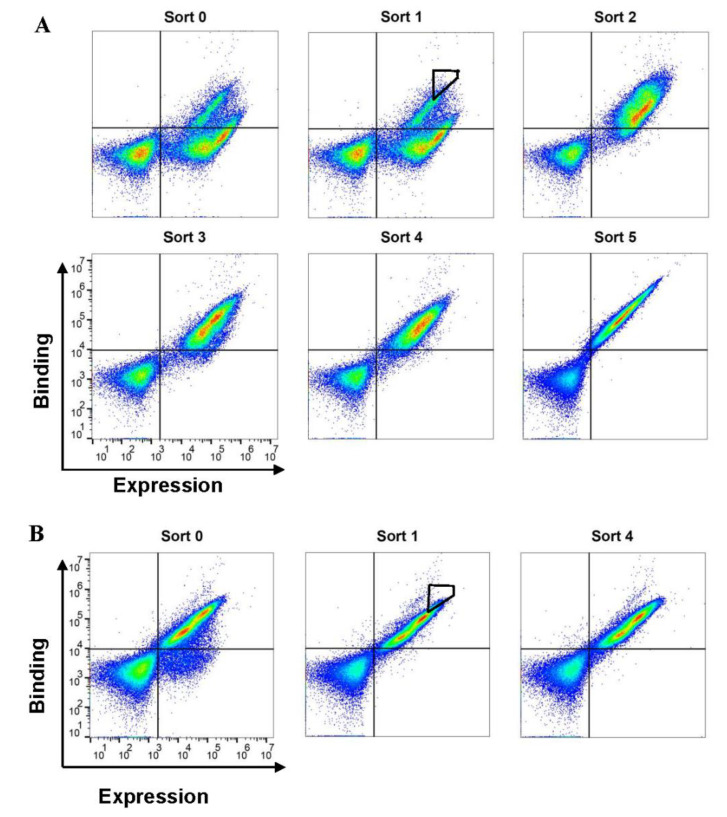
Flow cytometry sorting of yeast-display SCF_M_ library enriches the c-Kit binding population. Flow cytometry analysis of our first-generation (**A**) and second-generation (**B**) libraries. Yeast-displayed SCF was labeled with anti-c-myc antibody and secondary PE-conjugated antibodies (*x*-axis). Soluble c-Kit (added at 1 nM for analysis) was labeled with FITC (*y*-axis). Cells having variants with the highest affinities were sorted using the gates shown only for sort #1 in both panels for purposes of the illustration; the same gate was applied to all sorts.

Our strategy to generate SCF variants with different agonistic activities was based on modifying both SCF homo-dimerization and SCF binding affinity to c-Kit. We started by generating a double mutant variant, SCF_M_ V49L F63L, exhibiting reduced homo-dimerization [13]. We expressed this mutant on the surface of yeast, as a platform for affinity enrichment (Figure S1 in the Supplementary Material). The first yeast surface display (YSD) library based on SCF_M_ was then generated with an initial diversity of 4.5 × 10^6^. By using fluorescence-activated cell sorting (FACS), this library was enriched for clones with high expression and enhanced affinity for the soluble c-Kit extracellular domain. Six rounds of sorting were performed with decreasing concentrations of c-Kit (Figure 1A). Two SCF_M_ clones, designated SCF_M,K91E_ and SCF_M,S64P,F126S,V131A,E134G,V139I_, were chosen for purification based on their high frequency in the selected population and their enhanced affinity towards soluble c-Kit in the YSD system, respectively (Figure S2A). A second-generation library, based on SCF_M,K91E_, was then generated, with an initial diversity of 3 × 10^6^. This second library was further enriched through four rounds of sorting (Figure 1B), resulting in the identification of three high-affinity variants, designated SCF_M,K91E,K24N_, SCF_M,K91E,D97G_ and SCF_M,K91E,L98R_, with the last of these three being the most frequent mutant in the high-affinity sorting gates (Figure S2B). SCF_WT_, SCF_M_, SCF_M,K91E_, SCF_M,K91E,L98R_ and SCF_M,S64P,F126S,V131A,E134G,V139I_ were purified in non-glycosylated form, and their purity and molecular weights were determined (Figure S3). Circular dichroism (CD) spectroscopy showed that SCF_M_ and SCF_M,K91E_ shared the same secondary structure with SCF_WT_, according to our measurements and in agreement with a previous publication [33] (Figure S4A). Notably, CD spectra of the glycosylated and non-glycosylated SCF_WT_ revealed that protein glycosylation did not affect the global secondary structure of the protein (Figure S4A).

### 2.2. The Dimeric State of SCF Proteins is Concentration Dependent

**Figure 2:**
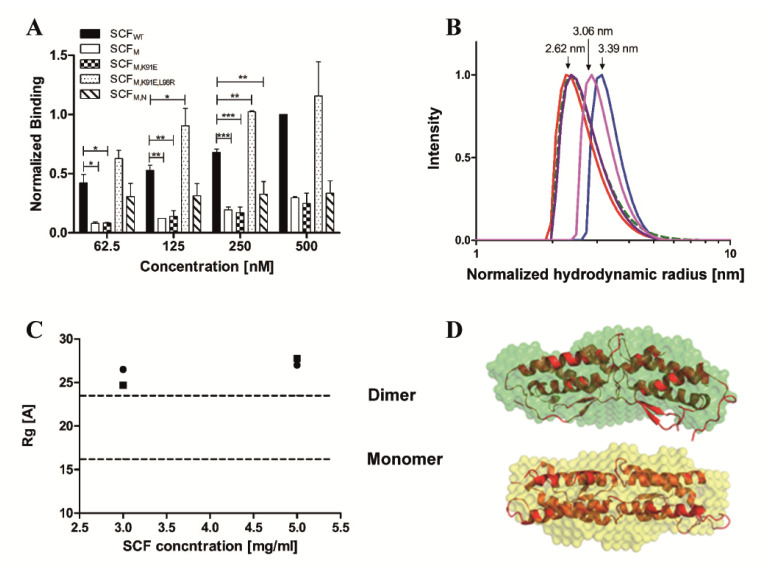
Mutations of SCF affect dimerization. (**A**) Homo-dimerization of soluble SCF proteins with the same yeast-surface displayed (YSD) SCF protein for: SCF_WT_ (black columns), SCF_M_ (white columns), SCF_M,K91E_ (checkered columns), SCF_M,K91E,L98R_ (dotted columns) and SCF_M,S64P,F126S,V131A,E134G,V139I_ (SCF_M,N_, cross-hatched columns). The values obtained for the binding of the soluble SCF proteins to YSD SCF proteins were normalized to the value that was obtained for the binding of soluble SCF_WT_ (at 500 nM) to the YSD SCF_WT_. Values are given as means of three independent experiments ± SEM, * *p* < 0.05; ** *p* < 0.01; *** *p* < 0.001. (**B**) DLS analysis of SCF_WT_ (blue), SCF_M_ (red), SCF_M,K91E_ (dashed green), SCF_M,K91E,L98R_ (pink), and SCF_M,S64P,F126S,V131A,E134G,V139I_ (purple). (**C**) SAXS results showing the radius of gyration (Rg) of SCF_WT_ (circles) and SCF_M_ (squares) that was measured at 3 and at 5 mg/mL. The Rg values for the theoretical SCF_WT_ monomer and dimer were calculated from the crystal structure (PDB: 1SCF) and are indicated as dashed lines. (**D**) The crystal structure of the SCF dimer was aligned with the SAXS models obtained for SCF_WT_ (green) and SCF_M_ (orange) using PyMOL.

Biophysical experiments were then performed to determine the extent of dimerization of each SCF protein. The association of the soluble form of each protein, SCF_WT_, SCF_M_, SCF_M,K91E_, SCF_M,K91E,L98R_, and SCF_M,S64P,F126S,V131A,E134G,V139I_, with the same SCF protein displayed on yeast was monitored (Figure 2A and Figure S1). In the concentration range of 62.5–500 nM (1 × 10^−3^–8 × 10^−3^ mg/mL), the dimerization of SCF_WT_ and SCF_M,K91E,L98R_ was stronger—by more than threefold—than that of SCF_M_, SCF_M,K91E_, or SCF_M,S64P,F126S,V131A,E134G,V139I_. We also used dynamic light scattering (DLS) to compare the molecular sizes (hydrodynamic radii) of these purified proteins. The hydrodynamic radius of SCF_WT_ (0.5 mg/mL; 32.25 µM) of 3.39 ± 0.57 nm was larger than the hydrodynamic radii of SCFM, SCFM,K91E and SCFM,S64P,F126S,V131A,E134G,V139I, having values of 2.62 ± 0.17, 2.77 ± 0.25 and 2.65 ± 0.69 nm, respectively (Figure 2B). The hydrodynamic radius of SCFM,K91E,L98R (3.06 ± 0.2 nm) was larger than the hydrodynamic radii of the above three variants, but smaller than that of SCF_WT_, suggesting that more SCF_WT_ and SCF_M,K91E,L98R_ units are in dimeric form than those of SCF_M_, SCF_M,K91E_, or SCF_M,S64P,F126S,V131A,E134G,V139I_ (Figure 2B). To gain additional independent information about the dimerization of soluble SCF, we calculated the radius of gyration (Rg) of SCF_WT_ and SCF_M_ by using small angle X-ray scattering (SAXS). The measured data indicated that both SCF_WT_ and SCF_M_ at concentrations of 3 or 5 mg/mL (193 or 322 µM, respectively), which are 10-fold higher than the concentrations used in DLS, are dimers (Figure 2C,D). For both proteins, the Rg value was slightly higher than the theoretical Rg of the SCF_WT_ dimer. Taken together, the above findings show that at low concentrations, SCF_WT_ forms dimers and SCF_M_ is monomeric, but at higher concentrations both proteins are dimers.

### 2.3. SCF_M,K91E_ and SCF_M,K91E,L98R_ Exhibit High Affinity for c-Kit

**Figure 3:**
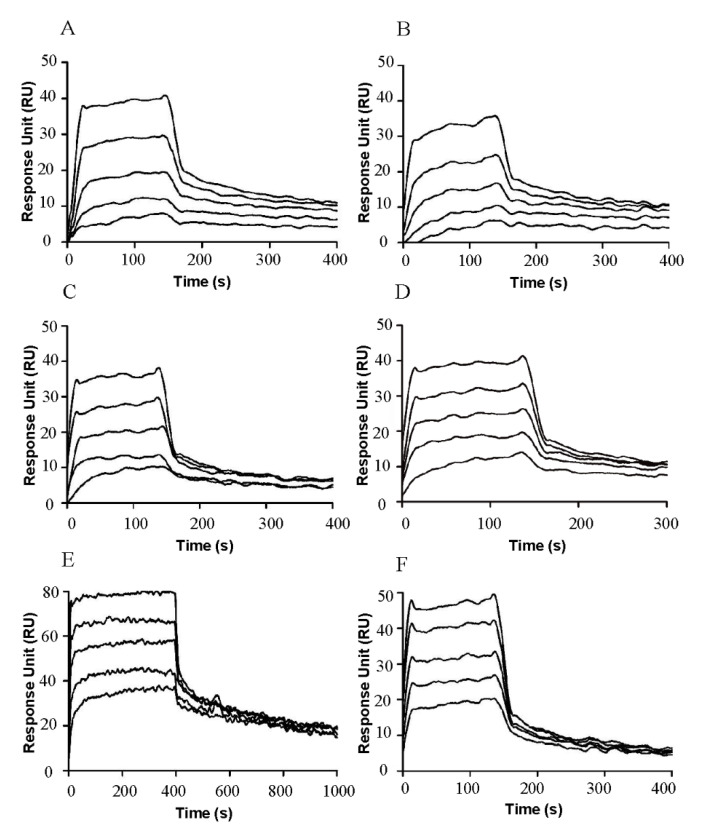
Affinities of SCF variants for c-Kit. The association and dissociation of the soluble SCF variants to and from surface-immobilized c-Kit. (**A**) Non-glycosylated SCF_WT_; (**B**) Glycosylated SCF_WT_; (**C**) SCF_M_; (**D**) SCF_M,K91E_; (**E**) SCF_K91E,L98R_; and (**F**) SCF_M,S64P,F126S,V131A,E134G,V139I_.

To directly measure the affinity of each SCF protein for c-Kit, we used surface plasmon resonance (SPR) with the receptor c-Kit protein immobilized on the chip and soluble ligand concentrations of 0.62–50 nM for glycosylated and non-glycosylated SCF_WT_, or 31.5–500 nM for SCF_M_, SCF_M,K91E_, SCF_M,K91E,L98R_ and SCF_M,S64P,F126S,V131A,E134G,V139I_ (Figure 3).

The K_D_ values for the glycosylated and non-glycosylated SCF_WT_ interacting with the immobilized c-Kit were very similar, namely, 5.82 ± 2.93 or 4.36 ± 1.46 nM, respectively (Table 1), with both being in the previously reported K_D_ range for the SCF/c-Kit interaction (0.5–65 nM) [35,45]. As expected, SCF_M_ showed a much lower affinity (K_D_ = 146 ± 18.3 nM) for c-Kit. The affinities of SCF_M,K91E_ and SCF_M,S64P,F126S,V131A,E134G,V139I_ were 88.9 ± 19.9 nM and 74.4 ± 5.09 nM, respectively. Interestingly, the affinity of SCF_M,K91E,L98R_ was 40.7 ± 0.7 nM, which is higher than that for the other mutants, but still lower than that of SCF_WT_.

**Figure 4:**
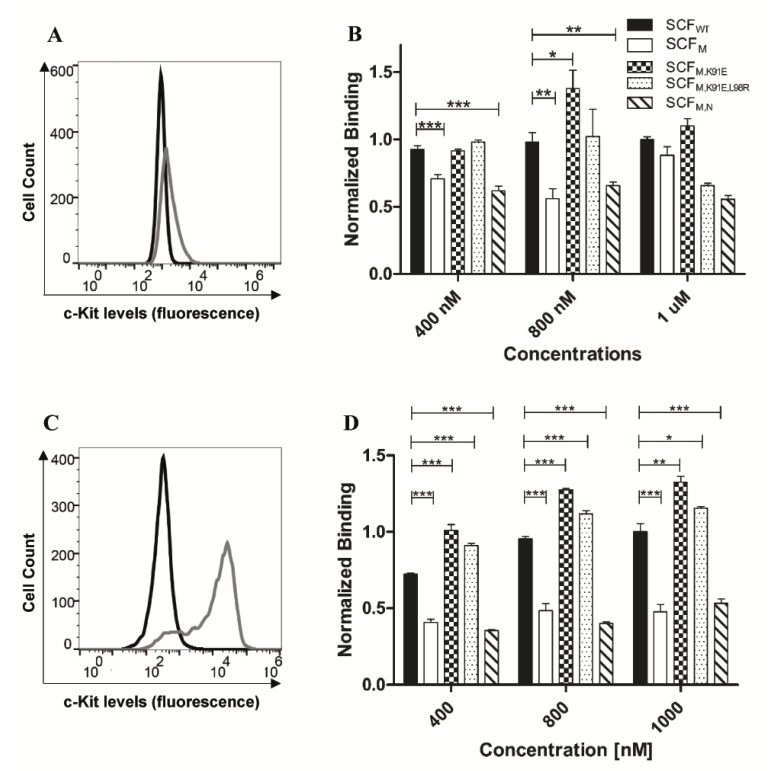
Improved and reduced binding of SCF variants to cell-surface c-Kit. (**A**) Expression levels of c-Kit in the A172 cell line. Grey and black lines represent c-Kit expression and background signals, respectively. (**B**) Binding of SCF variants to A172 cells expressing c-Kit. (**C**) Expression levels of c-Kit in murine HSPCs. (**D**) Binding of SCF variants to HSPCs. In panels (**B**,**D**) the columns are designated as follows: SCF_WT_ (black columns), SCF_M_ (white columns) and SCF_M,K91E_ (checkered columns), SCF_M,K91E,L98R_ (dotted columns) and SCF_M,S64P,F126S,V131A,E134G,V139I_ (cross-hatched columns). The results were normalized to SCF_WT_ binding at the highest concentration of 1000 nM. Values are means ± SEM of independent measurements performed in triplicate. * *p* < 0.05; ** *p* < 0.01; *** *p* < 0.001.

To further test the affinities of SCF_WT_, SCF_M_, SCF_M,K91E_, SCF_M,K91E,L98R_ and SCF_M,S64P,F126S,V131A,E134G,V139I_ for c-Kit, we evaluated their binding to c-Kit in two different cell lines, namely, A172, a brain glioblastoma cell line, and murine HSPCs. In agreement with the above-mentioned YSD and SPR binding results, SCF_M,K91E_ and SCF_M,K91E,L98R_ exhibited a higher affinity for the A172 cells, as compared to SCF_M_, reaching the effective binding levels of SCF_WT_. In contrast, SCF_M,S64P,F126S,V131A,E134G,V139I_ showed weaker binding to A172 cells, compared with SCF_WT_ (Figure 4A). Murine HSPCs showed high expression of the c-Kit receptor, which has 83% sequence homology with human c-Kit [35,46] (Figure 4C). These cells, too, showed SCF_M,K91E_ and SCF_M,K91E,L98R_ to exhibit the highest binding (Figure 4D), suggesting that SCF_M,K91E_ and SCF_M,K91E,L98R_ bind human or murine c-Kit with similar or even higher affinities compared to the parental SCF_M_. The conformation that c-Kit adopts when displayed on the membrane of intact cells vs. that when it is immobilized on the SPR sensor chip may be different for the different epitopes that are exposed and available for binding SCF_M,S64P,F126S,V131A,E134G,V139I_ vs. SCF_M,K91E,L98R_ and/or SCF_M,K91E_. These differences may result in a small discrepancy between the SPR and cell binding results.

### 2.4. SCF-Dimerization is the Major Determinant for c-Kit Phosphorylation in Human Umbilical Vein Endothelial Cells

**Figure 5:**
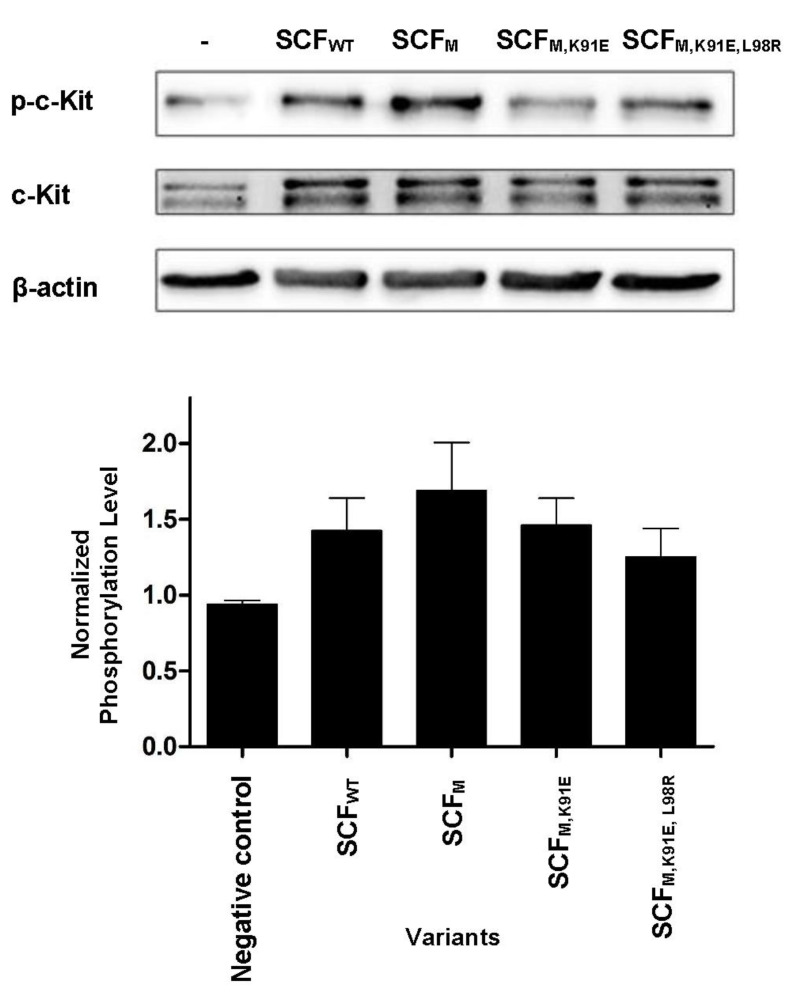
SCF variants activate c-Kit on primary human umbilical vein endothelial cells (HUVECs). HUVECs were incubated with SCF proteins (SCF_WT_, SCF_M_, SCF_M,K91E_ or SCF_M,K91E,L98R_) at a concentration of 250 nM. c-Kit phosphorylation levels were normalized to the expression levels of c-Kit (second row) and to β-actin levels (third row). Values are means ± SEM of independent measurements performed in triplicate.

**Figure 6:**
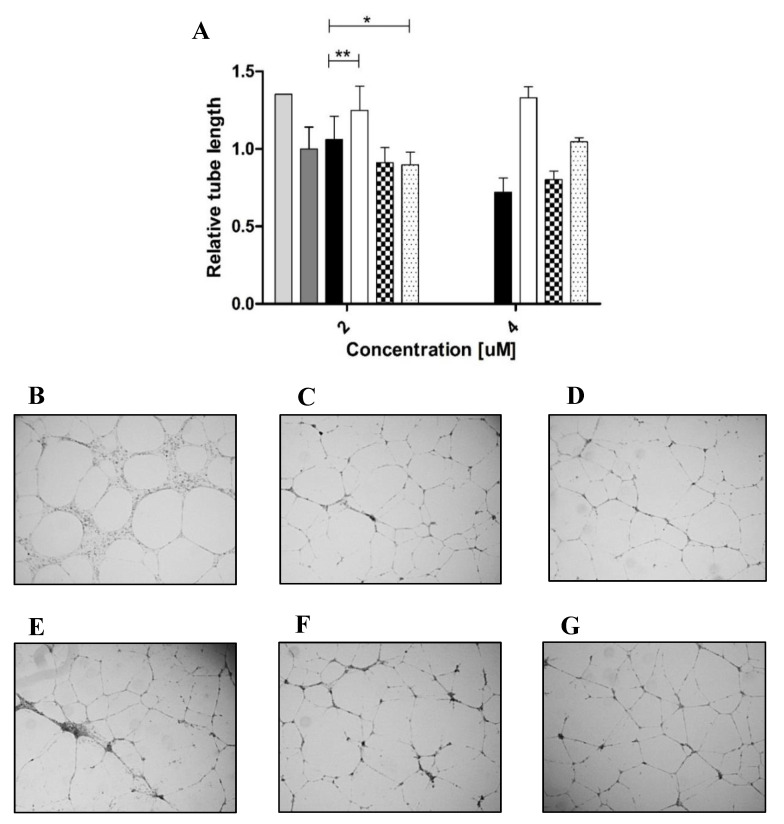
SCF variants with different functional effects on primary human umbilical vein endothelial cells (HUVECs). (**A**) HUVECs with 2% fetal bovine serum (FBS) as the positive control (light gray column), untreated HUVECs as negative control (dark gray column), and cells treated with 4 µM of SCF_WT_ (black columns), SCF_M_ (white columns), SCF_M,K91E_ (checkered columns) and SCF_M,K91E,L98R_ (dotted columns). Tube length was normalized to untreated cells, which served as a control. Values are means ± SEM of independent measurements performed in triplicate. (**B**) Positive control. (**C**) Negative control. (**D**) SCF_WT._ (**E**) SCF_M_. (**F**) SCF_M,K91E_. (**G**) SCF_M,K91E,L98R_. In panels (**B**–**G**) all proteins were added at a concentration of 4 µM. * *p* < 0.05; ** *p* < 0.01.

Stimulation of c-Kit by SCF activates a wide array of signaling proteins and pathways, starting with c-Kit phosphorylation and followed by activation of phosphatidylinositide 3′-kinase (PI3-kinase), Scr family kinases (SFK) and Ras-Erk pathways. In human umbilical vein endothelial cells (HUVECs), these events lead to cell proliferation and the formation of capillary-like structures by the endothelial cells [47]. To test for the activation of the c-Kit receptor by the different variants, we thus performed a phosphorylation assay in HUVECs as a cell-based model of angiogenesis. For this assay, we used concentrations of the same order as those used in the above-described biophysical and biochemical assays. As shown previously, these concentrations are representative of the local concentrations of SCF in tissues rather than of global levels in serum or other body fluids [48] (even though they are substantially higher than the concentrations used in some previous studies [13]). Western blot with a specific phospho-c-Kit showed that all the proteins induced receptor activation, but to different extents. The striking—and somewhat unexpected—finding was the difference between SCF_WT_ and SCF_M_: SCF_WT_ induced c-Kit activation by strongly binding to it as a dimer (Figure 5), but SCF_M_ bound to c-Kit as a monomer, being the only variant to do so. Furthermore, although the affinity of SCF_M_ for c-Kit was lower than that of SCF_WT_, SCF_M_ was the only protein that increased c-Kit activation to a greater (albeit modestly) extent than SCF_WT_. Thus, the SCF_WT_ homo-dimer and SCF_M_ monomer acted in a similar way as c-Kit agonists, with the latter being slightly more potent in activating c-Kit expressed in HUVECs, as shown by its ability to induce the HUVECs to assemble into tube-like structures (Figure 6). In contrast, SCF_M,K91E_ and SCF_M,K91E,L98R_ were inactive in this assay.

## 3. Discussion

This study of engineered SCF ligands and their interaction with c-Kit, the cognate SCF receptor, provides proof of concept that receptor-activating ligands can be modulated to confer different agonistic potencies through changes of affinity for the cognate receptor and dimerization of the ligand itself. The study also showed that enhancement of the agonistic potency of SCF was concentration dependent in that the concentration in our study was very high, being in the micromolar range vs. the nanomolar range of previous studies [13]. Currently, most studies on SCF are conducted with concentrations in the range of 3–100 ng/mL; this concentration range, which is clearly appropriate for the routine growth of cells, does not represent the actual local concentration at the cell surface. The naïve-state concentration of SCF in the circulation is 3.3 ng/mL; thus, the active concentration would certainly be higher and could reach substantially higher local concentrations at the cell surface [48]. Since it was our intention to test for robust biochemical properties of the studied mutants, we used a concentration range that would represent more faithfully the high local concentrations of SCF.

Reducing SCF dimerization while preserving or even enhancing its affinity for c-Kit will change the dynamics of binding and activation. At high concentrations, namely, at concentrations representative of tissue levels, SCF_WT_ tends to dimerize before binding to c-Kit, with the SCF_WT_ dimer, with its two binding sites, being likely to approach a single c-Kit receptor molecule. One binding site of the SCF_WT_ dimer will therefore bind to one c-Kit molecule, while the other will remain free. In such a scenario, it is likely that most or all of the c-Kit molecules are each bound to a different SCF_WT_ dimer via a single SCF monomer such that the dimerization of SCF_WT_ (bound to c-Kit) and the subsequent c-Kit dimerization and activation would be slow or unlikely. In contrast, at low (nanomolar) SCF_WT_ concentrations, as in previous studies [13], this dimeric ligand may bind two c-Kit molecules at the same time, resulting in c-Kit dimerization and activation. A different scenario is likely for the SCF_M_ variants. SCF_M_ will first bind as a monomer to c-Kit, and then dimerize with another SCF_M_ protein attached to another c-Kit receptor, thereby bringing two c-Kit receptors into proximity and promoting c-Kit dimerization and hence activation. This scenario may explain the increase of c-Kit activation by the monomeric SCF_M_ at the high concentrations tested (250 nM in the phosphorylation assay and 2 µM and 4 µM in the tube formation assay). In addition, intermediate levels of c-Kit activation could be obtained by enhancing the affinity of a mutated SCF_M_ ligand for its c-Kit receptor, as was the case for SCF_M,K91E_ and for especially SCF_M,K91E,L98R_, in order to compensate for changes in ligand dimerization, as these two characteristics are not mutually exclusive.

To identify SCF_M_ mutants with enhanced affinity for c-Kit, random mutations were introduced, followed by screening yeast surface displayed SCF_M_ libraries against c-Kit and selection of the mutants with the highest affinity in increasingly stringent sorts. The fact that during affinity maturation the SCF_M_ proteins were displayed as monomers on the surface of yeast was important, since it meant that mutagenesis was controlled in such a way that affinity enhancement was biased towards dimerization or a higher order of multimerization. Indeed, sequencing of various clones selected from the final sorting round for the first- and second-generation libraries yielded several unique sequences, all including V49L and F63L mutations, that conferred enhanced affinity of SCF_M_ variants for c-Kit. We found that the K91E mutation observed in the affinity-engineered SCF_M_ clones (i.e., SCF_M,K91E_ and SCF_M,K91E,L98R_) was near the SCF hot-spot residues related to the interaction of SCF with c-Kit and not to SCF-SCF dimerization (Supplementary Materials: Figure S5). These hot-spot residues may be involved in improving the affinity between SCF and c-Kit, either through direct binding interactions or by structural stabilization. Using SPR spectroscopy, we identified SCF variants with up to 3.7-fold higher affinity for recombinant c-Kit than the parental SCF_M_, even though the variants and SCF_M_ possess similar receptor binding sites. SPR spectroscopy also showed that glycosylation of SCF_WT_ did not influence its affinity for c-Kit, suggesting SCF/c-Kit interactions take place through the amino-acid backbone of SCF without a direct role of post-translational modifications, such as glycosylation.

It was found that the SCF_M,K91E_ and SCF_M,K91E,L98R_ variants exhibited improved binding to A172 cells vs. SCF_M_. Strikingly, SCF_M,K91E_, and to an even greater extent SCF_M,K91E,L98R_, also showed stronger binding than the dimeric SCF_WT_ to the murine primary cells. It is thus likely that the L98R mutation may be involved in improving SCF homo-dimerization such that the monomeric SCF_M,K91E_ possesses only a single receptor binding site, whereas SCF_M,K91E,L98R_ possesses two c-Kit receptor binding sites and thereby promotes an enhancement of SCF-c-Kit interactions. Similarly, the ability of the variants to induce c-Kit activation and to activate the formation of capillary-like structures in HUVECs to different extents could be explained by differences in both their ability to self-associate (with SCF_M_ and SCF_M,K91E_ binding as monomers to c-Kit, and SCF_WT_ and SCF_M,K91E,L98R_ binding as dimers) and in their affinity for recombinant soluble c-Kit or cell-surface-expressed c-Kit (with SCF_M,K91E,L98R_ and SCF_MK91E_ having higher affinity for c-Kit than SCF_M_).

Another factor contributing to the different effects of the mutant proteins may lie in their physical state: DLS measurements showed that SCF monomer self-association to a dimer was prevented for SCF_M_ and SCF_M,K91E_, but not for SCF_WT_ and SCF_M,K91E,L98R_, which did form dimers. Perhaps more importantly, SAXS measurements, which were performed to obtain low-resolution structures of SCF_WT_ and SCF_M_ at high concentrations in solution, demonstrated that the two proteins are similar in size and shape, both of which are compatible with a dimeric structure of SCF [49]. The mutations in the monomeric SCF_M_ (i.e., V49L and F63L) appeared to significantly decrease its affinity for purified and cell-expressed c-Kit relative to SCF_WT_. This is presumably due to decreased avidity, as at low concentrations SCF_M_ cannot bind two c-Kit molecules. Importantly, at high concentrations (3 or 5 mg/mL), SCF_M_ can dimerize. In contrast, the mutation K91E in SCF_M,K91E_ appears to maintain this variant in its monomeric state, while increasing its affinity for both purified and cell-expressed c-Kit relative to SCF_M_. The mutation L98R in SCF_M,K91E,L98R_ compensates for the V49L and F63L mutations and restores SCF homo-dimerization at low concentrations. These biophysical observations can therefore explain the order of potency in the early and late signaling biological events (i.e., phosphorylation and tube formation, respectively) of the proteins: At high concentrations, SCF_M_ is a monomer in solution prior to binding and becomes a dimer upon enhancement of its local concentration after binding to c-Kit expressed on cells. This makes SCF_M_ a strong activator of c-Kit. SCF_WT_ dimerizes better at low concentrations, such that it binds c-Kit strongly as an SCF–SCF dimer, probably capturing both binding sites (one on each monomer) on a c-Kit dimer. As a result, at the high SCF concentrations tested here, c-Kit activation following SCF_WT_ self-association and c-Kit dimerization, is weaker than that with SCF_M_, but at low SCF concentrations the reverse is true, as shown in previous studies [13]. SCF_M,K91E,L98R_, for example, binds c-Kit as a c-Kit dimer and thus has an effect that is similar to that of SCF_WT_.

Our approach complements other methods for targeting SCF for therapeutic application. A recent example of that type of study was presented by a group from Novartis [50] demonstrating the potency of a c-KIT-directed ADC (a humanized anti-c-KIT antibody conjugated to a microtubule destabilizing small molecule) in models of mutant and wild-type c-KIT-positive solid tumors. In this respect, the significance of our study stems from the insight it provides into the sequence-structure–function relationships and mechanism of action of agonistic and antagonistic SCF mutants and it will therefore support engineering of further improved SCF variants as potential therapeutics. Moreover, the approach of using a natural protein ligand as a molecular scaffold for engineering high affinity agents can be applied to other ligands and to create functional protein agonists and antagonists against additional biomedical targets of interest.

## 4. Materials and Methods

### 4.1. Generating Random Mutagenesis SCF_M_ Libraries in Yeast

The gene encoding for SCF_M_ was designed on the basis of SCF_WT_ with two mutations, one at V49L and the other at F63L [13]. The gene was purchased from Integrated DNA Technologies (IDT, Coralville, IA, USA) and amplified by PCR with a Pfx50 polymerase (Life Technologies, Carlsbad, CA, USA). The PCR product and pCTCON (a generous gift from the laboratory of Dane Wittrup, MIT) [38] vector were digested with the restriction enzymes BamHI and Nhe1, ligated using T4 ligase (New England Biolabs, Ipswich, MA, USA), according to a standard protocol, and transformed into competent EBY100 *Saccharomyces cerevisiae* yeast cells using a MicroPulser electroporator (Bio-Rad, Hercules, CA, USA). The plasmid was extracted from the yeast using a Zymoprep™ yeast plasmid miniprep I kit (Zymo Research, Irvine, CA, USA). A first-generation DNA library originating from SCF_M_ (used as a template for error prone PCR) was constructed using error prone PCR with low fidelity Taq DNA polymerase (New England Biolabs) and 2 uM of 8-oxo dGTP and dPTP nucleotide analogs (Jena Bioscience, Jena, Germany). The primers with pCTCON homology used for library preparation were TAAGGACAATAGCTCGACGATTGAAG and GATTTTGTTACATCTACACTGTTG. A second amplification (to generate a second-generation library) used Phusion DNA polymerase (New England Biolabs) with SCF_M,K91E_ as a template DNA and the primers GTTCCAGACTACGCTCTGCAGG and CAGATCTCGAGCTATTACAAG. Cloning into pCTCON was performed according to the same protocol as that used to produce the first-generation library. These reactions allowed homologous recombination of the inserts (PCR products) and the pCTCON vector. The yeast library was grown in SDCAA medium (2% dextrose, 0.67% yeast nitrogen base, 0.5% Bacto™ Casamino acids, 1.47% sodium citrate, 0.429% citric acid monohydrate, pH 4.5) at 30 °C with shaking at 300 rpm, until the culture reached OD_600_ = 10.0 (10^8^ cells/mL).

### 4.2. Screening of SCF Libraries

Yeast cells expressing each library were grown in SGCAA medium (2% galactose, 0.67% yeast nitrogen base, 0.5% Bacto Casamino acids, 1.47% sodium citrate, 0.429% citric acid monohydrate) overnight at 30 °C, with shaking at 300 rpm, until the culture reached OD_600_ = 10. Cells were washed with PBSA 1% [phosphate buffered saline (PBS) with 1% bovine serum albumin (BSA)]. The cells were double labeled with 1:50 anti c-myc antibody (9E10, Abcam, Cambridge, UK) and different concentrations of human recombinant c-Kit-Fc (Abcam) in PBSA 1% for 1 h at room temperature. Cells were washed with 1% PBSA and incubated with a sheep anti-mouse antibody conjugated to phycoerythrin (PE) (Sigma Aldrich, Rehovot, Israel) and a goat anti-human Fc conjugated to fluorescein isothiocyanate (FITC) (Sigma Aldrich, Rehovot, Israel), both at a 1:50 ratio for 30 min on ice in the dark. The first-generation library was sorted multiple times using SY3200 FACS (Sony Biotechnology, Bothell, WA, USA), and the second-generation library was sorted multiple times using FACS ARIA III (BD Biosciences, San Jose, CA, USA). For each sort round, the desired population was enriched by collecting the high-expressing (PE-labeled) and high c-Kit-binding (FITC-labeled) clones by using sorting gates that included 0.3–3.5% of the entire cell population. For affinity maturation of the SCF libraries, in each sort the c-Kit concentration was sequentially reduced. After each sort, the library was labeled according to the same protocol and analyzed using Accuri C6 flow cytometer (BD Biosciences) and FlowJo software (Tree star Inc., Ashland, OR, USA). SCF_M_ individual clones (20 to 40) from each sort were sequenced (as above) using Geneious R7 (Biomatters, Auckland, New Zealand). The binding of individual SCF variants (in their YSD format) to soluble c-Kit was analyzed (as before), and SCF_WT_, SCF_M_, SCF_M,K91E_, SCF_M,K91E,L98R_, and SCF_M,S64P,F126S,V131A,E134G,V139I_ were chosen for production and purification as described in the Supplementary Materials.

### 4.3. Dimerization Assays Using Flow Cytometry

Yeast cells expressing SCF proteins (SCF_WT_, SCF_M_, SCF_M,K91E_, SCF_M,S64P,F126S,V131A,E134G,V139I_ and SCF_M,K91E,L98R_) were induced overnight in SGCAA medium, washed with 1% PBSA, and aliquoted at 1 × 10^6^ cells/sample. Cells displaying the SCF proteins were labeled either with anti c-myc or with different concentrations of purified proteins (SCF_WT_, SCF_M_, SCF_M,K91E_, SCF_M,K91E,L98R_, and SCF_M,S64P,F126S,V131A,E134G,V139I_) in 1% PBSA for 2 h at 4 °C on a rotary shaker. For secondary staining, PE-conjugated sheep anti-mouse and allophycocyanin (APC)-conjugated anti-Flag (BioLegend, San Diego, CA, USA) were used. Values are presented as mean fluorescence ± SEM; statistical significance was determined using a *t*-test. *p* value < 0.05 was considered statistically significant.

### 4.4. Dynamic Light Scattering

DLS was used to determine the hydrodynamic radius of the purified proteins (SCF_WT_, SCF_M_, SCF_M,K91E_, SCF_M,K91E,L98R_, and SCF_M,S64P,F126S,V131A,E134G,V139I_) in PBS in concentrations ranging between 0.3–0.5 mg/mL. Proteins were centrifuged for 1 h at 10,000 rpm and filtered through a 0.22-µm filter to remove contaminants. Spectra were collected with a CGS-3 goniometer (ALV, Munich, Germany). The laser power was 20 mW at the helium-neon laser line (633 nm). Correlograms were calculated by the ALV/LSE 5003 correlator, which were collected 10 times, each time over 20 s, at 25 °C. The analysis was repeated four times at the detection angles of either 90° or 60°, in independent measurements. The correlograms were fitted by the CONTIN program [51]. DLS signal intensity was transformed to number distribution based on the Stokes–Einstein equation [52].

### 4.5. Small Angle X-ray Scattering

SAXS data were collected on SAXLAB GANESHA 300 XL system, possessing a Genix 3D Cu-source, an integrated monochromator, three-pinhole collimation, and a two-dimensional Pilatus 300K detector. SCF_WT_ and SCF_M_ were measured at concentrations of 3 or 5 mg/mL. A buffer-only sample was used to set the background. The measurements were performed under vacuum at 25 °C. The scattering vector (*q*) ranged between 0.012 and 0.7 Å^−1^. The magnitude of the scattering vector is described by $q=\frac{4\pi \;sin\theta }{\lambda }$, where 2*θ* is the scattering angle and *λ* is the wavelength. The values for the radius of gyration (*Rg*) were derived from a Guinier plot, namely, a linear small-angle part of the SAXS scattering curve (*qRg* < 1.0), in PRIMUS. In this region, the Guinier approximation is applicable:

$$I\left(q\right)=I\left(0\right)e^{\frac{-R_{g}^{2}q^{2}}{3}}$$

where *I* is the scattering intensity and q is the scattering vector magnitude (a function of the scattering angle). *Rg* values were also derived using internal scripts [53] designed to perform an automatic search for the best fitting parameters using GNOM [54]. CRYSOL [55] was used to compute the artificial SAXS spectra based on the available crystal structure of SCF 1–141 (PDB: 1SCF) [49]. These spectra served as a reference for the reconstruction of the experimental SAXS data. The molecular envelope was reconstructed by GASBOR [54] based on the best GNON fit achieved from the internal script.

### 4.6. Surface Plasmon Resonance

The affinity constant describing the interactions between c-Kit and soluble SCF proteins was determined on a ProteOn XPR36 instrument (Bio-Rad, Hercules, CA, USA). Recombinant c-Kit (R&D Systems) was immobilized on one channel of a ProteOn GLC sensor chip using the amine coupling reagents sulfo-NHS (*N*-hydroxysuccinimide; 10 mM) and EDC (1-ethyl-3-(3 dimethylaminopropyl)-carbodiimide; 40 mM) (Bio-Rad, Hercules, CA, USA). c-Kit, 1.2 μg or 2 μg, was covalently immobilized on the chip in 10 mM sodium acetate buffer, pH 4.0, to give 3866 or 5237 response units (RU), respectively. BSA (3 μg; 4706 RU) was immobilized on the chip as a negative control on a different channel. Unbound esters were deactivated with 1 M ethanolamine HCl at pH 8.5. The temperature was set at 25 °C, and the proteins were then allowed to flow over the chip in a range of concentrations (i.e., in series of threefold dilutions from 50 nM to 0.6 nM) for the glycosylated and non-glycosylated SCF_WT_ and in a series of twofold dilutions from 500 nM to 31.25 nM for the remainder of the proteins (SCF_M_, SCF_M,K91E_, and SCF_M,S64P,F126S,V131A,E134G,V139I_). All the proteins were dissolved in PBST solution (PBS 0.005% Tween 20) and were then allowed to flow over the SPR chip surface at 100 μL/min for 150 s, followed by a dissociation phase of 270 s in PBST. After each run, a regeneration step was conducted with 50 mM NaOH. For each protein complex, a sensogram was generated from the RUs measured during the course of the protein–protein interactions by subtracting the background response of flow cells immobilized with BSA. The dissociation constant (K_D_) was determined from a sensogram of the equilibrium-binding phase. The data were analyzed with ProteOn manager 3.1.0.6 and fitted to 1:1 Langmuir binding model. To achieve statistical significance, only measurements with *χ*^2^ values that were at least 12% or lower than the Rmax values were chosen for analysis [56].

### 4.7. Cell Binding Assays

The A172 glioblastoma cell line was grown in Dulbecco’s modified Eagle’s medium (DMEM, Biological Industries, Beit HaEmek, Israel) with 10% FBS, 1% L-glutamine (Biological Industries) and 1% penicillin streptomycin (Biological Industries, Beit-Haemek, Israel). Cells were harvested with trypsin, and distributed to give 100,000 cells/well. Volumes of 100 µL containing different concentrations of each SCF protein were incubated in the different wells for 2 h at 4 °C on a rotary shaker. Cells were washed twice and then stained with APC conjugated anti-FLAG (BioLegend, San Diego, CA, USA) for 30 min on ice in the dark. The cells were analyzed using Accuri C6, and the data was analyzed using FlowJo software.

Using the same protocol, FACS analysis was performed on murine HSPCs from bone marrow. Lineage^−^c-Kit^+^Sca1^+^ bone marrow primary cells were grown in BioTarget medium (Biological Industries, Beit HaEmek, Israel) with 1% L-glutamine, 1% penicillin streptomycin, 10 ng/mL SCF, thyroid peroxidase (TPO), IL-3, and FLT-3 (Peprotech, Rehovot, Israel). The cells were analyzed using Gallios flow cytometer (Beckman Coulter Inc., Carlsbad, CA, USA), and the data was analyzed by Kaluza (Beckman Coulter Inc., Carlsbad, CA, USA) and Prism software (GraphPad, San Diego, CA, USA). The results are presented as means ± SEM of triplicate measurements. Statistical significance was determined using *t*-test analysis. *p* value < 0.05 was considered statistically significant.

### 4.8. c-Kit Phosphorylation

HUVECs were grown in EGM-2 medium (Lonza, Basel, Switzerland) containing serum and growth factors^40^. Cells were grown in 6-well plates to 80% confluence and starved for 18 h by incubation in EBM-2 medium without serum and growth factors. Cells were stimulated for 7 min with 250 nM of each of the SCF proteins (i.e., SCF_WT_, SCF_M_, SCF_M,K91E_ and SCF_M,K91E,L98R_). The cells were washed twice with cold PBS and collected in 200 µL of lysis buffer [50 mM Hepes, 150 mM NaCl, 10% glycerol, 1% Triton X-100, 1.5 mM MgCl_2_, 1 mM EDTA, 1 mM Na_3_VO_4_ and protease inhibitor cocktail (Roche)] for 10 min on ice. The samples were clarified by centrifugation at 15,000 rpm for 10 min at 4 °C. The quantity of total proteins in the lysed cells was determined using a BCA kit (Thermo Scientific, Waltham, MA, USA). Western blot used 20 µg of proteins per lane. After blocking the membrane with TBST (50 mM Tris-HCl, pH 7.4, 150 mM NaCl, 0.1% Tween 20) supplemented with 5% BSA, primary antibodies [i.e., rabbit-anti-phospho-c-Kit (R&D Systems, Minneapolis, MN, USA) at a dilution of 1:2500; rabbit-anti-c-Kit (R&D Systems, Minneapolis, MN, USA) at a dilution of 1:1500; or β-actin antibody (Cell Signaling Technology, Danvers, MA, USA) at a dilution of 1:1000] were added for an overnight incubation at 4 °C. The membrane was washed three times for 10 min with TBST, and then a secondary antibody, i.e., anti-rabbit IgG-HRP (Cell Signaling Technology, Danvers, MA, USA), at a dilution of 1:1000 was added for 1 h at room temperature. Blots were developed with EZ-ECL Kit (Biological Industries, Beit HaEmek, Israel). The bands were visualized by chemiluminescence with Fusion-FX7 spectra (Vilber Lourmat, Collégien, France), and the intensity of each band was measured with the image analysis ImageJ software. Values are given as means ± SEM of triplicates.

### 4.9. Matrigel Endothelial Tube Formation Assay

Growth factor reduced (GFR) Matrigel (Corning, New York, NY, USA) was used to coat a 96-well plate by centrifugation at 300× *g* for 10 min at 4 °C and incubation at 37 °C for 30 min. HUVEC cells (3.5 × 10^4^ cells per well) were resuspended in EBM-2 with different concentrations of each SCF protein. After incubation of 16–18 h at 37 °C, the plate was monitored with an EVOS FL Cell Imaging System (Thermo Scientific, Waltham, MA, USA), at a ×2 magnification. The results were analyzed with ImageJ software and with Prism software (GraphPad, San Diego, CA, USA). Values are given as means ± SEM of quadruplicates.

## 5. Conclusions

In summary, the overall goal of this research was to use both rational and combinatorial synthetic methods to develop a new generation of SCF-derived proteins as c-Kit agonists with potential therapeutic applications and as tools to study basic ligand–receptor recognition and receptor activation during key biological processes, in this case SCF-SCF interactions and their influence on SCF/c-Kit function. We found that reduced dimerization of the SCF variants prior to receptor binding resulted in enhanced c-Kit receptor activation, whereas improved receptor binding affinity of the engineered SCF monomers resulted in a reduced receptor activation. In seeking explanations for our findings, we fully characterized the binding and biological properties of the purified variants by employing cell-based models of receptor activation. Our data shows that the impairment of SCF dimerization may increase the local SCF concentration near c-Kit and thereby induce enhanced dimerization and activation of the receptor.

## Figures and Tables

**Table 1:** Binding affinities of SCF variants. The dissociation constant (K_D_) was determined from SPR sensograms of the equilibrium-binding phase. K_D_ values are means ± SD of three independent experiments.

Protein	K_D_ (M)
SCF_WT_	4.36 × 10^−9^ ± 1.46 × 10^−9^
SCF_WT_ glycosylated	5.82 × 10^−9^ ± 2.93 × 10^−9^
SCF_M_	146 × 10^−9^ ± 18 × 10^−9^
SCF_M,K91E_	88.9 × 10^−9^ ± 19.9 × 10^−9^
SCF_M,K91E,L98R_	40.7 × 10^−9^ ± 0.7 × 10^−9^
SCF_M,S64P,F126S,V131A,E134G,V139I_	74.4 × 10^−9^ ± 5.1 × 10^−9^

## Supplementary Materials

The following are available online, Figure S1: YSD; Figure S2: Binding analysis of individual SCF variants to soluble c-Kit; Figure S3: Protein production and purification; Figure S4: CD spectra of the purified proteins; Figure S5: Schematic representation showing the locations of SCF mutations in the SCF/c-Kit complex (PDB: 1SCF).
