# Supplementary material for: Engineering Stem Cell Factor Ligands with Different c-Kit Agonistic Potencies
Source: Molecules. 2020 Oct 21;25(20):4850. doi: 10.3390/molecules25204850 (PMC7588011; doi:10.3390/molecules25204850)
Supplement: Supplementary file 1 [file molecules-25-04850-s001.pdf]

Engineering Stem Cell Factor Ligands with Different c-Kit Agonistic Potencies

Tal Tilayov[1,*], Tal Hingaly[1,*], Yariv Greenshpan[2], Shira Cohen[3], Barak Akabayov[3], Roi Gazit[2], and Niv Papo[1,**]

[1]Avram and Stella Goldstein-Goren Department of Biotechnology Engineering and the National Institute of Biotechnology in the Negev, Ben-Gurion University of the Negev, Beer-Sheva, Israel; taltil@gmail.com; talhingaly@gmail.com; papo@bgu.ac.il
[2]Shraga Segal Department of Microbiology, Immunology and Genetics and the National Institute of Biotechnology in the Negev, Ben-Gurion University of the Negev, Beer-Sheva, Israel; yarivg@post.bgu.ac.il; gazitroi@bgu.ac.il
[3]Department of Chemistry and the National Institute for Biotechnology in the Negev (NIBN), Ben-Gurion University of the Negev, Beer-Sheva, Israel; shiracoh@post.bgu.ac.il; akabayov@bgu.ac.il

*These authors contributed equally to this work.
**Correspondence: papo@bgu.ac.il; Tel: +972-50-2029729.

Materials and Methods

Protein purification

The genes encoding for the SCF variants (SCF_{WT}, SCF_{M}, $SCF_{M,K91E}$, $SCF_{M,K91E,L98R}$, and $SCF_{M,S64P,F126S,V131A,E134G,V139I}$) were amplified from pCTCON vector by PCR. The reaction was performed with Phusion DNA polymerase (New England Biolabs, USA) using as forward primer GCCGAGGAATTCGAAGGTATTTGTAGAAAC and as reverse primer CCCTACCCTAGGTGATACAACGCAATCTG, except for $SCF_{M,S64P,F126S,V131A,E134G,V139I}$ in which the reverse primer was GCCGCGCCTAGGTGATACAATGCAATCT. The PCR product was digested with EcorI and AvrII (New England Biolabs, USA) and ligated to a recombinant pPIC9K plasmid (Invitrogen, Israel) containing the AOX1 promotor for expression the genes. The pPIC9K plasmid contains an N-terminal Flag epitope tag and a C-terminal 6×His-tag for labeling and purification of the expressed protein, respectively. The ligated plasmid was then transformed into *Escherichia coli* cells, as described in the Materials and Methods section, and sequenced. The plasmids were linearized using SacI restriction enzyme (New England Biolabs, USA) and transformed into a competent *Pichia pastoris* GS115 strain (Invitrogen, Israel) by using the Multi-Copy Pichia expression kit protocol (Invitrogen, Israel). After transformation, the yeast was grown on RDB plates (18.6 % sorbitol, 2 % agar, 2 % dextrose, 1.34 % yeast nitrogen base, 4×10^{-5} % biotin and 5×10^{-3} % each of L-glutamic acid, L-methionine, L-leucine, L-lysine, and L-isoleucine) to recover and then collected and plated on a YPD selective medium with Geneticin plates (4 mg/ml Geneticin). A few colonies were picked randomly and cultured in 5 mL of BMGY (2 % peptone, 1 % yeast extract, 0.23 % $K_2H(PO_4)$, 1.1812 % $KH_2(PO_4)$, 1.34 % yeast nitrogen base, 4×10^{-5} % biotin, 1% glycerol) overnight at 30 °C in order to identify a high expression clone for each variant. Each clone was induced by resuspending each culture in 5 mL of BMMY (2 % peptone, 1 % yeast extract, 0.23 % $K_2H(PO_4)$, 1.1812 % $KH_2(PO_4)$, 1.34 % yeast nitrogen base, 4×10^{-5} % biotin, 0.5% methanol) for 3 days at 30 °C; methanol was added each day in a final concentration of 0.5%. On the third day, western blot analysis was performed using 20 μL of cells taken from each culture. Mouse anti-Flag primary antibody (Sigma Aldrich, Israel) at a ratio of 1:000 followed by anti-mouse antibody conjugated to alkaline phosphatase (Jackson ImmunoResearch, West Grove, PA, USA) at a ratio of 1:5000 were incubated for 1 h at room temperature. BCIP regent (2 mL, Sigma Aldrich, Israel) was used for analysis. The clone from the culture with the highest protein expression level was further selected for a large-scale production, which included inoculation into 50 mL of BMGY and overnight incubation at 30 °C, followed by 3 days of incubation in 500 mL of BMMY. During this period, methanol was added each day to a final concentration of 0.5%. On the third day, the culture was prepared for purification by Ni/NTA affinity chromatography. For this purification step, the culture was centrifuged and filtered, and then NaCl (final concentration of 500 mM) and imidazole (final concentration of 10 mM) were added. Thereafter, the supernatant was equilibrated to pH 8.0 and allowed to stand for 1 h at 4 °C. The supernatant was

centrifuged and filtered again. The supernatant was then uploaded onto a Ni/NTA column (GE Healthcare Life Sciences, USA) overnight by using a peristaltic pump. The column was washed with a buffer containing 20 mM sodium phosphate, 500 mM NaCl and 10 mM imidazole, pH 8.0, and then eluted with buffer containing 20 mM sodium phosphate, 500 mM NaCl and 500 mM imidazole, pH 8.0. The protein buffer was then replaced with PBS, and the solution was concentrated by using Vivaspin with a 5-kDa cutoff (Vivaproducts, MA, USA). Where needed, sugars linked via N-glycosylation sites were removed from the concentrated protein by using 1:10 G5 buffer and 2 μL of Endo-HF enzyme (New England Biolabs) overnight at 25 °C. All proteins were further purified using AKTA pure 150 FPLC with a Superdex 75 10/300 size-exclusion chromatography column (GE Healthcare Life Sciences, USA) with elution buffer (50 mM Tris, pH 7.5, 100 mM NaCl and 5 mM $CaCl_2$). During the purification process, the proteins were loaded on 15% SDS-PAGE to evaluate their purity. The yield of each of the purification processes was 0.5-3 mg per 1 L of yeast culture, as determined using a NanoDrop spectrophotometer (Thermo Scientific, MA, USA) (extinction coefficient 13,200 $M^{-1}cm^{-1}$), based on protein absorbance at 280 nm. Protein samples were also subjected to mass spectrometry analysis (Ilse Katz Institute for Nanoscale Science and Technology, Ben-Gurion University of the Negev, Israel).

Circular dichroism (CD) analysis

Structure determination of the proteins was performed using J-815 spectropolarimeter (JASCO) in a 1-mm path quartz cuvette. Spectra of 50 μM purified protein (SCF_{WT}, SCF_{M}, $SCF_{M,K91E}$ or $SCF_{M,S64P,F126S,V131A,E134G,V139I}$) in PBS, pH 7.2, were obtained at room temperature over the range of 185-260 nm for structure determination. Each spectrum was a merge of 4 scans and was normalized to obtain ellipticity (degree × cm^2/dmol).

Supplementary Figures

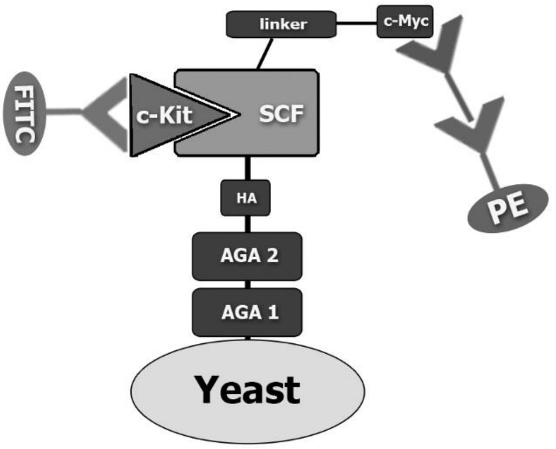

Figure S1. YSD. Schematic representation of the yeast surface display (YSD) system. SCF proteins were displayed on the yeast surface and the soluble target was c-Kit. A primary antibody labeled with FITC and a secondary labeled with PE were used for detection of binding to c-Kit and SCF mutant expression on the yeast surface, respectively. This diagram also presents the experimental setup for the flow cytometry assay in which the association of the soluble form of each protein, SCF_{WT}, SCF_{M}, $SCF_{M,K91E}$, $SCF_{M,K91E,L98R}$, and $SCF_{M,S64P,F126S,V131A,E134G,V139I}$, with the same SCF protein (SCF in the rectangular box) displayed on yeast was monitored. AGA 1 and AGA2 stand for cell wall anchoring subunits of the a-agglutinin heterodimer.

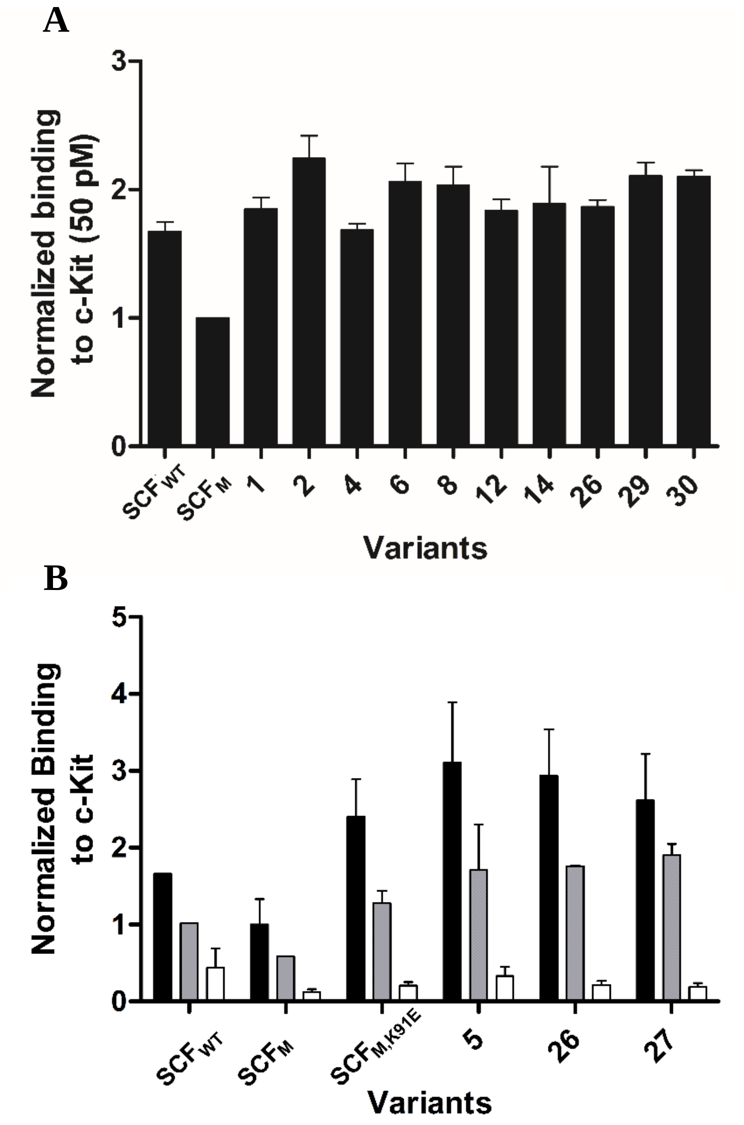

Figure S2. Binding analysis of individual SCF variants to soluble c-Kit. (A) Affinity of SCF_{WT} and first-generation YSD SCF_{M} library variants for c-Kit. Mean fluorescence affinity values were obtained by flow cytometry analysis using 50 pM c-Kit. The identity of individual clones is indicated on the X-axis as a number. (B) Affinity of second generation YSD SCF_{M} library variants for c-Kit. Mean fluorescence affinity values were obtained by flow cytometry analysis using different concentrations of c-Kit (black: 100 nM, gray:10 nM, white:1 nM). The identity of individual clones in panel B is indicated on the X-axis and corresponds to 5 – $SCF_{M,K91E,K24N}$, 26 – $SCF_{M,K91E,D97G}$, and 27– $SCF_{M,K91E,L98R}$. Binding values were normalized to SCF_{M} binding values tested against 100 nM c-Kit. Values are means ± SEM of three independent experiments.

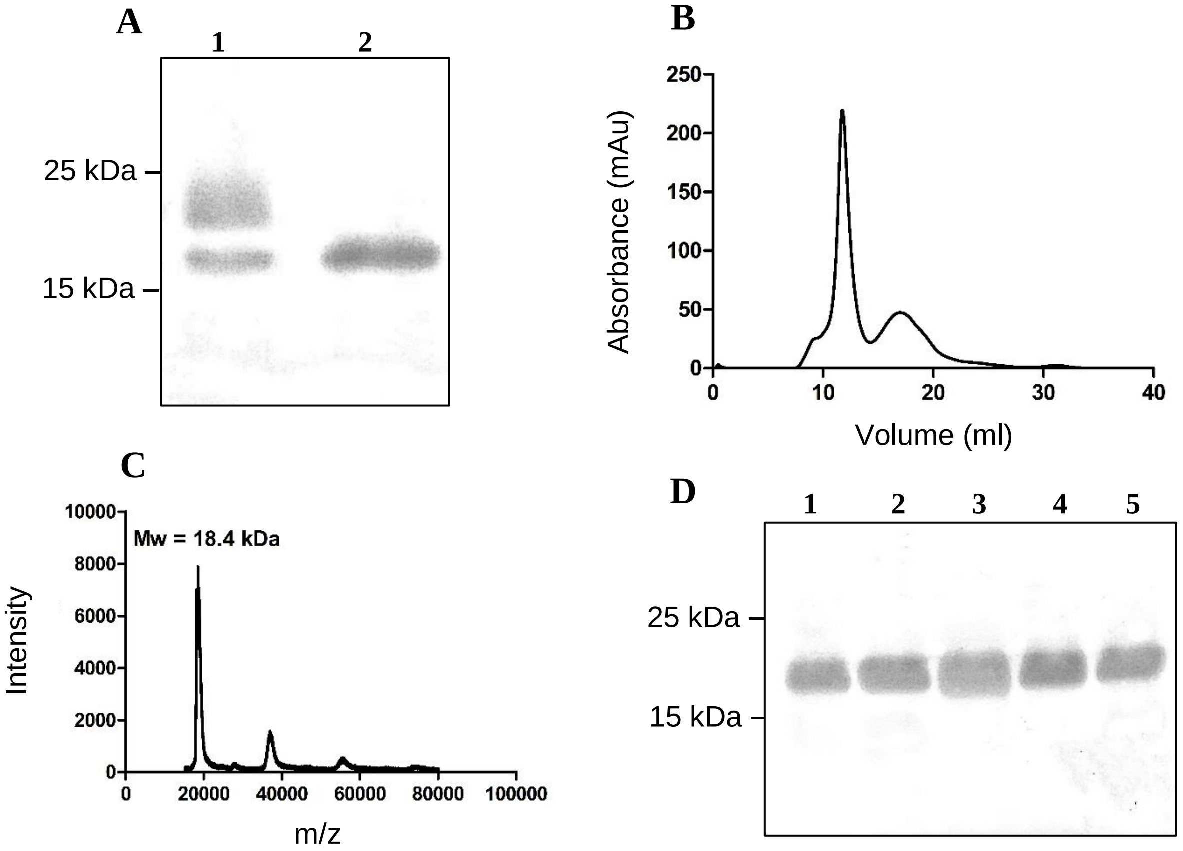

Figure S3. Protein production and purification. (A) SDS-PAGE of SCF$_{M,K91E}$ post Ni/NTA affinity chromatography (lane 1) and after deglycosylation followed by size-exclusion chromatography (SEC) (lane 2). (B) SEC analysis of SCF$_{WT}$. The same chromatogram was obtained for the other SCF proteins. (C) Mass spectrometry results for SCF$_{M,K91E}$. (D) SDS-PAGE for purified variants: lane 1 - SCF$_{WT}$, lane 2 - SCF$_{M}$, lane 3 - SCF$_{M,K91E}$, lane 4 - SCF$_{M,S64P,F126S,V131A,E134G,V139I}$, lane 5 - SCF$_{M,K91E,L98R}$.

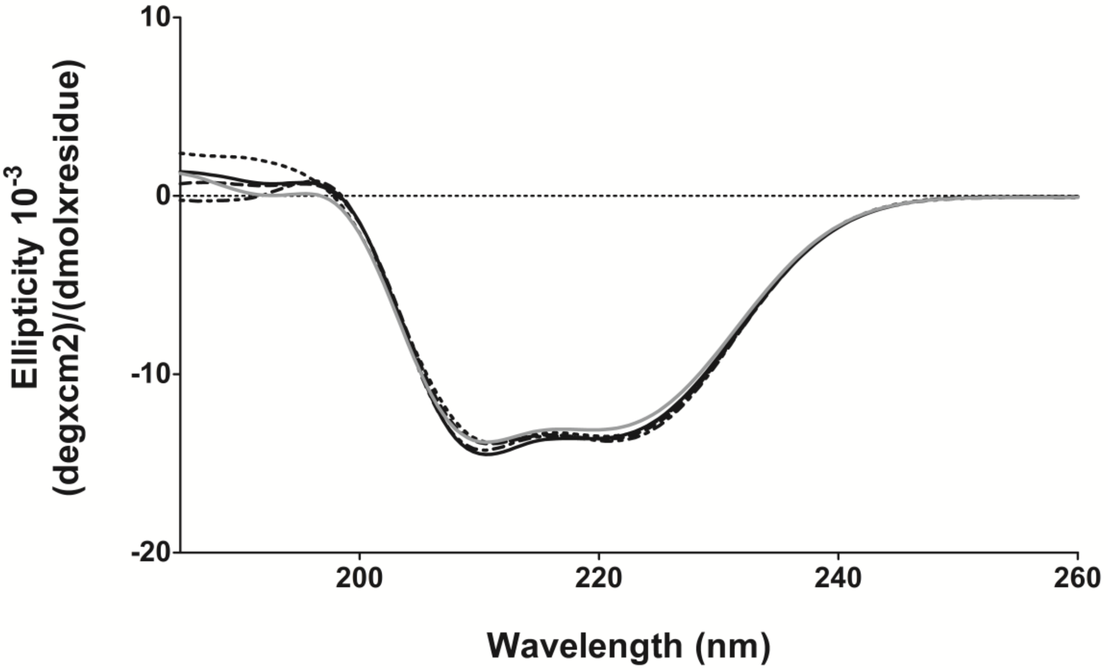

Figure S4. CD spectra of the purified proteins. (A) Structure analysis of non-glycosylated SCF_{WT} (black solid line), glycosylated SCF_{WT} (solid gray line), SCF_{M} (black small lines) $SCF_{M,K91E}$ (black dots) and $SCF_{M,S64P,F126S,V131A,E134G,V139I.}$ (black dot and line). All measurements were performed with 50 μM protein in PBS, pH 7.4, at 25 °C.

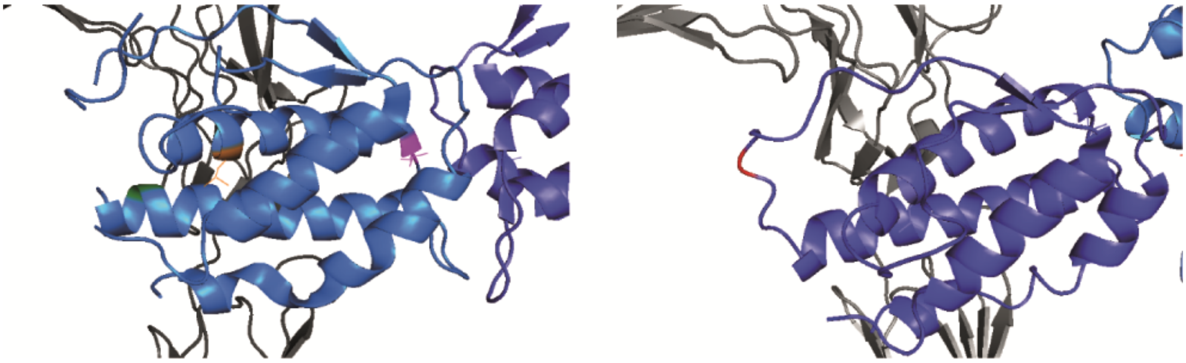

Figure S5. Schematic representation showing the locations of SCF mutations in the SCF/c-Kit complex (PDB: 1SCF). Mutations V49L, F63L, K91E and L98R are highlighted in brown, purple, green and red, respectively. SCF is shown in blue, and c-Kit, in grey.
